# Misdiagnoses of Epilepsy as Ekbom Syndrome, Mood Instability, and Nocturnal Visual Hallucinations

**DOI:** 10.1155/2017/3968751

**Published:** 2017-07-13

**Authors:** M. Duarte Mangas, Y. Martins, L. Bravo, A. Matos Pires

**Affiliations:** Psychiatry Service, Hospital José Joaquim Fernandes, Beja, Portugal

## Abstract

Epileptic seizures may be misdiagnosed if they manifest as psychiatric symptoms. We report three female patients with no preexisting history of epilepsy that were unsuccessfully treated as primary psychiatric disorder: one patient was initially diagnosed with somatization and Ekbom syndrome; the second was referred to psychiatrist due to mood instability and visual hallucinations; and the third one was referred for anxiety and hallucinations related to sleep. A carefully taken medical history clarified diagnoses of epilepsy. None of the patients responded to medications aimed at treating psychiatric symptoms, and all the patients had favorable response to antiepileptic treatment. These cases illustrate that epileptic patients may experience nonconvulsive seizures that might be misdiagnosed as primary psychiatric disorder.

## 1. Introduction

Chadwick (1994) recommends defining an epileptic seizure as “an intermittent, stereotyped disturbance of consciousness, behavior, emotion, motor function or sensation that on clinical grounds is believed to result from cortical neuronal discharge” [1]. This definition conveys three important principles: (i) the core presenting feature, the seizure, is a transient abnormality of neurological function that is highly uniform from one episode to the next; (ii) the diagnosis depends primarily on clinical judgment; and (iii) the underlying mechanism of an epileptic seizure is an abnormal cortical discharge.

A great variety of epileptic presentations can meet criteria for psychiatric disorders, including brief psychotic disorder, generalized anxiety, major depressive disorder, dementia, and other conditions among the mental illness. Additionally, epilepsy and psychiatric disorders are not mutually exclusive diagnoses and frequently coexist [2]. Epileptic phenomena associated with some experiences or presentations similar to psychiatric symptoms may be misinterpreted as primarily mental disorder.

## 2. Case Presentation

### 2.1. Case 1

A 40-year-old Romanian woman, housewife, was referred to the psychiatry outpatient service by general practitioner, with a history of depressed mood, disturbed sleep for the past four years, and complaints that she had a snake inside her body. Her symptoms initially led to diagnosis of anxiety, somatization, and depression. She was treated in primary care with antidepressants, antipsychotics, and anxiolytics, with no response. Her medical history included gastritis, a distal left leg fracture with three surgical interventions (last one five years ago), and a pituitary microadenoma which had been revealed by brain computerized tomography- (CT-) scan but was not confirmed by posterior brain magnetic resonance imaging (MRI). Blood works, including endocrine tests, were normal. At the psychiatric evaluation, the patient was conscious, cooperative, and anxious. She had slow, hesitant speech due to language barrier, however a coherent one. She reported “a snake squeezing around her body, starting in her left leg and spreading to the rest of her body, up to the neck.” This occurred during night time and did not let her sleep. No other delusions or perceptions alterations were revealed. The Ekbom syndrome was considered as a diagnostic hypothesis and the patient was medicated with risperidone and trazodone. Patient returned to the second assessment with no improvements. She was slightly irritable and she verbalized frustration since the treatment showed no result. After careful reconstruction of clinical history, a further detailed analysis of her complaints was performed. Finally, the patient said that she had complained of a feeling like a snake squeezing her body, but not of a true snake. When questioned, the patient described a feeling of something crawling under the skin and a feeling of pins and needles that started in her distal left leg, spread to left thigh, left hemibody, and left side of her neck. The symptoms occurred every night and lasted minutes. No change of consciousness was reported by the patient or by her husband, who has observed several episodes per night. The diagnosis of somatosensory Jacksonian seizures was considered. An anticonvulsant ex juvantibus treatment with levetiracetam was started. In two weeks, the symptoms were greatly reduced in intensity and frequency, allowing the patient to sleep. An EEG study was prescribed but yet not performed due to patient's departure to Romania. The patient was referred to neurologist for further observation and etiological clarification of her epilepsy.

### 2.2. Case 2

A 55-year-old woman, teacher, was referred to psychiatrist by general practitioner who described her symptoms as hallucinations and mood instability with frequent crying and laughing. She was treated with various antidepressants and herbal medications with no response. Her medical and familiar history was otherwise unremarkable. A brain CT-scan was normal.

At the psychiatric evaluation, the patient was conscious and cooperative; her speech was coherent and her mood was euthymic. During analysis of her complaints, she reported that, since her thirties, she had visual and auditory hallucination, saw images of “teeth laughing,” and heard some sounds described as “noises without any sense that I can't understand,” which lasted up to 5 minutes. The patient added that she also cried and laughed frequently, but she did not associate this with changes of her mood or with any other reason. She described it as an uncontrolled behavior, mentioning “it's a stupid laugh.” The patient was accompanied by her boyfriend, who described short laughing and crying episodes, through a few minutes, with no apparent reason. During the initial assessment, we observed two spontaneous crying episodes that lasted 1-2 minutes, with no reason.

EEG and conventional MRI were asked. Antiepileptic treatment with oxcarbazepine was prescribed.

Within a month, the patient was reevaluated with positive response to treatment; the symptoms had remitted completely. Cerebral MRI was within normal limits. Routine EEG showed unspecific slow activity (theta wave) predominantly in frontotemporal area of the left hemisphere.

The patient was referred to neurologist for further assessment.

### 2.3. Case 3

A 63-year-old woman, housewife, was referred to psychiatric service by general practitioner due to anxiety disturbance and nocturnal visual hallucinations. The patient was medicated with antidepressants and anxiolytics without benefit. Her medical history included diabetes mellitus, hypertension, hyperlipidemia, thyroid goiter, and cervical disc hernia. There was no relevant family history or drug abuse.

Routine EEG, cerebral CT, and analysis (including thyroid function) were normal.

At the psychiatric evaluation, the patient was conscious, oriented, and cooperative. She was anxious, with an apprehensive facial expression. The speech was coherent and focused on her health problems and on her difficulty of having a good night sleep.

When asked to detail her complaints, it emerged that patient's symptoms had started at the age of 35 years. She mentioned “visual hallucinations during her sleep” of which she only remembered that “there was something terrible.” Her husband mentioned that every night, during the sleep, the patient suddenly became agitated. He described screaming, fear, bizarre movements of feet and legs, more rarely, movements of upper extremities and, very rarely, complex motor behavior as getting out of bed with posterior falling down (no generalized seizures observed), and perplexity if awakened. These episodes were stereotypic and lasted about 5–10 minutes. No aggressive behavior was observed. No eventual trigger factors were identified.

The patient also mentioned that in daytime she felt anxious due to the fear of falling asleep and repeating the same symptoms; that is why she complained of her “fear to be alone.”

An anticonvulsant treatment with oxcarbazepine was started. Within one week, symptoms remitted completely, allowing her to a good night sleep, which the patient considered an unequivocal improvement. She still mentioned anxiety and fear of being alone; for that, an antidepressant (escitalopram in low dose) was added. One month later, no night episodes occurred; anxiety symptoms also remitted.

The patient was referred to neurologist for further observation, exams, and etiological clarification.

## 3. Discussion

In the three cases presented, all patients experienced epileptic seizures during years and received psychiatric treatment with no response. The diagnosis of epilepsy was clinical, supported by significant improvement with antiepileptic ex juvantibus treatment. Although no specific lesions were detected by CT or MRI, if possible, all patients, even seizure-free, should continue to be studied for identification of seizure's etiology.

The first case describes a partial somatosensory seizure, with typical* march* starting at distal leg region and spreading to ipsilateral side of body and neck. The language barrier and the specific description of the symptoms by the patient as a “snake” led to initial misinterpreting of clinical picture as delusional infestation (Ekbom syndrome). The depressive symptoms appear to be reactive to successive therapeutic failures. This case is also interesting per se due to clinical pattern of seizures, always developed from the site of leg fracture and started around 11 months after the last surgery. We found some rare case reports describing focal epilepsy developed after soft tissue lesions of hands, with start of seizures in the injured hand. It is presumed that the peripheral injuries can result in plastic changes in cortex causing hyperexcitability and seizures [3].

The second patient presented gelastic and dacrystic seizures interpreted as mood changes. Gelastic seizures are mainly attributed to hypothalamic hamartomas; however it is also reported that epileptogenic zone may be in the hypothalamus as well as in the temporal lobe. In some cases, this type of seizure may be also a manifestation of epilepsy of other localizations or multifocal epilepsy [4, 5]. The phenomenology of seizures experienced by our patient (concomitant visual and auditory hallucination) suggests involvement of other regions, such as temporal lobe. In our opinion, this patient, although seizure-free, should be studied for identification of seizure's etiology.

In the third case, the patient's behavior was interpreted as hallucinations, although she did not remember their content. This case required differential diagnosis between sleep-related hypermotor epilepsy (SHE) and nonepileptic movement disorders in sleep, mainly REM sleep behavior disorder, NREM parasomnias (or disorders of arousal), and nightmares [6]. It is not easy to clinically distinguish these conditions, but according to criteria they are differentiated by [7] age at onset; no history of sleep disorders in childhood and adolescence; high frequency of episodes; absence of trigger factors; highly stereotypic motor pattern; no violent behavior; evolution to complex motor behavior with falling down; and impairment of consciousness. The aforementioned allowed us to consider SHE (not necessarily originated from the frontal lobe) as the most probable diagnosis hypothesis. For a definitive diagnosis conclusion, a video-EEG-polysomnography should be performed. The anxiety symptoms seem to be reactive and were resolved after the improvement of night episodes and with a low dose of antidepressant.

## 4. Conclusion

Epilepsy includes a variety of neuropsychiatric symptoms. These cases illustrate that epileptic patients may experience nonconvulsive seizures that might be misdiagnosed as primary psychiatric disorders. Psychiatrists must be aware of these varied presentations while obtaining the medical history in order to investigate and manage these patients effectively.
